# Incidence of and Risk Factors for Dementia in the Ibadan Study of Aging

**DOI:** 10.1111/j.1532-5415.2011.03374.x

**Published:** 2011-05-13

**Authors:** Oye Gureje, Adesola Ogunniyi, Lola Kola, Taiwo Abiona

**Affiliations:** * Department of Psychiatry, University of Ibadan, University College HospitalIbadan, Nigeria; † Department of Neurology, University of Ibadan, University College HospitalIbadan, Nigeria

**Keywords:** incident dementia, community dwelling, developing country

## Abstract

**OBJECTIVES:**

To describe the incidence of dementia in a representative sample of elderly Yoruba Nigerians and provide information about the risk factors.

**DESIGN:**

In-home face-to-face assessments conducted on a community cohort selected using multistage clustered sampling of households, with baseline between November 2003 and August 2004 (n = 2,149) and follow-up approximately 39 months later (n = 1,408).

**SETTING:**

Eight contiguous, predominantly Yoruba-speaking states in Nigeria.

**PARTICIPANTS:**

Persons aged 65 and older free of dementia at baseline (n = 1,225).

**MEASUREMENTS:**

Dementia was ascertained using two instruments: the 10-Word Delayed Recall Test and the Clinician Home-based Interview to assess Function, both with demonstrated validity and cultural applicability.

**RESULTS:**

At 3-year follow-up, 85 participants had developed dementia. With a total 3,888 risk years for the sample, the estimated incidence of dementia was 21.85 per 1,000 person-years (95% confidence interval = 17.67–27.03). Compared with men, the age-adjusted hazard ratio (HR) for women was 2.12 (*P* = .002). Incidence increased linearly with age such that, compared with participant aged 65 to 74, the HR, adjusted for sex, for participants aged 75 to 84 was 2.84 (*P*<.001) and for those aged 85 and older was 4.13 (*P*<.001). Greater incidence of dementia was found with more-rural residence and poorer economic status. Participants with poor social engagement at baseline were at significantly greater risk of incident dementia.

**CONCLUSION:**

Incident dementia in Yoruba Nigerians is higher than previously reported. Indices of social isolation are risk factors for incident dementia in this population.

Even though dementia is a major health problem in old age and has become a cause of considerable public health concern, especially in developed countries, there is still a considerable gap in knowledge about the extent of the problem in developing countries.^1^–^3^ This is paradoxical, given that the majority of older adults in the world reside in developing countries and that projections suggest that, by 2040, 71% of the people with dementia in the world will reside in developing countries.^4^

Previous studies of dementia in developing countries have found widely varying rates of occurrence, with reported prevalence estimates ranging from 1.8% to 21.1%.^1^ Within this wide spread, a pattern of relatively low rates can be discerned from studies conducted in India and sub-Saharan Africa.^1^–^6^ For example, data derived from the longitudinal Indianapolis–Ibadan Dementia Project, have shown that the incidence of dementia is lower in older Yoruba Nigerians than in African Americans living in Indianapolis.^4^ In general, it was found that, although the incidence of dementia in African Americans was in the higher range of previous published incidence rates, that of the Yoruba was among the lowest. The question as to whether that low rate was peculiar to the cohort studied in that report or is generalized to the wider Yoruba ethnic group remains an open one. A report of a screening survey for dementia in which a prevalence estimate for probable dementia of approximately 10% was found provided an indication that the pattern may be different in the larger Yoruba community.^7^

Even though several lifestyle, social, and health-related risk factors, such as social engagement, physical activity, body mass index, and chronic health conditions such as hypertension, have been identified for dementia, data on the salience of these factors for the African population is sparse. Earlier reports showing that the previously demonstrated association in non-African populations between apolipoprotein E and dementia did not hold in Nigerian subjects is indicative of the need to make no a priori assumptions about the relevance of these reported risk factors for that population.^8^,^9^

## METHOD

### Sample

**Figure 1:**
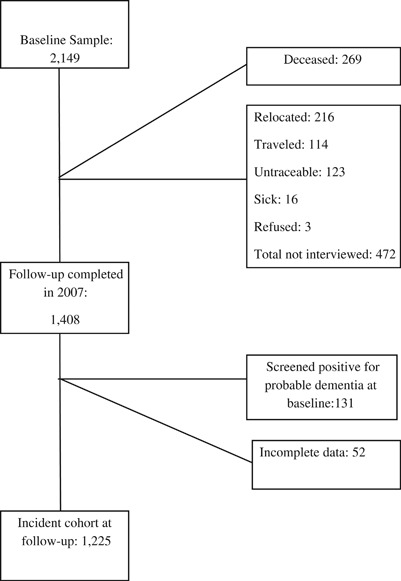
Study flow-chart.

The Ibadan Study of Aging (ISA) is a community study of the profile and determinants of healthy aging. A full description of the baseline methodology, conducted between November 2003 and August 2004, has been provided elsewhere.^7^,^10^,^11^ A clustered multistage random sampling of households was made to select a representative sample of noninstitutionalized older adults (aged ≥65). The resulting cohort of 2,149 with full data was followed up in 2007. Of the 2,149, 1,408 (65.5%) were successfully followed up approximately 3 years later; 269 were dead, 472 could not be traced, had traveled, or had moved (453); were too sick to be examined (16); or refused to be interviewed (3). The result of the follow-up exercise is shown in Figure 1.

### Instruments

#### Assessment of Dementia

Screening for dementia was conducted using the 10-Word Delayed Recall Test (10-WDRT). The 1-WDRT, adapted from the Consortium to Establish a Registry of Alzheimer's Disease 10-word learning list,^12^ is test of memory. It has been shown to be a valid tool, largely unaffected by educational level, for the assessment of dementia in Nigeria.^13^ A score threshold of <2, which has been shown, using data from previous studies,^4^,^5^ to have good psychometric indices for the identification of probable dementia, was used.^7^

The 10-WDRT was used to screen for possible dementia at baseline and follow-up. At follow-up, a second-stage assessment was conducted using the Clinician Home-based Interview to assess Function (CHIF).^14^ The CHIF, a 10-item semistructured home interview that evaluates respondent's higher cognitive function by assessing their knowledge of how to perform instrumental activities of daily living (IADLs), was developed to provide a reliable assessment of function in older adults. It collects information about personal history, cooking and food preparation, shopping, finances, medicines, religious attendance, communication with friends and relatives, social and family roles, organization of home and personal clothing, and recognition and awareness. Each item is scored from 0 to 2, with 2 indicating good performance. In the Ibadan site of the Indianapolis–Ibadan Dementia Project, the CHIF has an interrater reliability of 0.87 and a Cronbach alpha value of 0.83 for internal consistency. A test of its concordance with *Diagnostic and Statistical Manual of Mental Disorders, Fourth Edition* (DSM-IV) diagnosis of dementia based on comprehensive physician assessment, yielded an area under the receiver operating characteristic curve of 0.925, confirming its utility as a valid tool for the identification of dementia.^14^ With a score range of 0 to 20, a cutoff of <18 provides the best trade-off between sensitivity (89.5%) and specificity (68.5%).

A test of executive function, the animal fluency test, in which the number of different animals a respondent could name in 1 minute was counted, was also conducted.

#### Subject Assessment

Baseline assessment of dementia was conducted using the 10-WDRT, and no second-stage evaluation was done. A diagnosis of probable dementia was made at baseline in persons who scored less than 2 on the 10-WDRT. At follow-up, the 10-WDRT and the CHIF were administered to all respondents. Different interviewers conducted the two assessments independently, usually within 48 hours of one another. The 10-WDRT was the first to be administered as part of an interview lasting, on average, approximately 1 hour. Research supervisors (blinded to the 10-WDRT performance) administered the CHIF during an assessment that included measuring blood pressure, height, and weight, among others. At follow-up, a psychiatrist reviewed all available information to determine the presence or absence of dementia. The information included scores on the 10-WDRT and CHIF, the interviewer's observations (of respondent's memory and language, which the interviewers recorded at the end of each respondent's assessment), reported functional status (as obtained in several sections of the interview and commonly supplemented by key informant's report^10^), and the temporal relationship of the onset of any co-occurring depressive disorder. Although the CHIF obtained information of respondent's higher cognitive function by assessing their knowledge of how to perform instrumental ADLs, role functioning was evaluated to determine difficulties in the performance of ADLs and instrumental ADLs.^10^ The psychiatrist used all of the information in making a determination of presence or absence of dementia.

The validity of the diagnosis of dementia was tested by comparing persons with and without the diagnosis on their performance on the animal-naming fluency test included in the assessment protocol. The respective mean scores were 10.5 (95% CI = 8.9–12.2) and 13.3 (95% CI = 13.0–13.6), showing distinct, nonoverlapping score profiles. This pattern was also true for the comparisons of the groups subdivided on the basis of whether they had some level of formal education.

Estimates of incident dementia are presented in persons with no dementia at baseline (scored ≥2 on the 10-WDRT), who thereby constituted the incident cohort at follow-up.

### Baseline Risk Factors

Diagnosis of depression at baseline (and follow-up) according to the DSM-IV^15^ was made after an assessment conducted using the World Health Organization Composite International Diagnostic Interview, Version 3.^16^

At baseline, respondents were asked whether a physician had ever diagnosed them with diabetes mellitus, hypertension, or stroke. A measurement of mid-upper arm circumference, which has been shown to be a sensitive and valid measure of nutritional status in older Africans, with a value of 22.1 cm or less being indicative of moderate to severe undernutrition,^17^ was made on every respondent.

Social engagement was assessed using items from the World Health Organization Disability Assessment Schedule, version 2.^18^ The relevant items asked “During the last 30 days, how much did you join in family activities such as eating together, talking with family members, visiting family members, working together?” and “During the last 30 days, how much did you join in community activities such as festivities, religious activities, talking with community members, working together?” Answers are rated as 1 (not at all), 2 (a little bit), 3 (quite a bit), and 4 (a lot). For this article, responses to each of the items were dichotomized as not at all versus all others. Thus, persons who answered not at all to either of these measures were regarded to have had poor social engagement at baseline.

Respondents' physical activity was assessed using the International Physical Activity Questionnaire, a widely used tool with demonstrated cross-cultural validity.^19^ The questionnaire measures physical activity across all domains of leisure-time, work, transportation, and household tasks. The summary indicator was used to categorize the respondents into three levels of physical activity: low (physically inactive), moderate, and high levels of physical activity. These categories were based on standard scoring criteria (http://www.ipaq.ki.se).

The University of Ibadan/University College Hospital, Ibadan joint ethical review board approved all aspects of the study.

### Analysis

Follow-up assessments were conducted an average of 39.3 months (95% confidence interval (CI) = 39.1–39.5) after baseline interviews. Incidence rates were calculated by dividing the number of cases with onset of dementia in each group of interest by the number of person-years of observation in that group. The person-years at risk for an individual with dementia were calculated as the midpoint between baseline and the follow-up time. Incidence rates were calculated within sex groups and three age categories (65–74, 75–84, ≥85). The 95% CIs around the incidence rates were estimated by assuming the Poisson distribution. Female:male ratios for the rates were calculated using Cox proportional hazards analysis adjusted for baseline age, and the age group ratios were adjusted for sex.

Baseline risk factors for incident dementia were explored using logistic regression, and the results are presented as odds ratios (ORs) (adjusted for age, sex, and educational level) with 95% CIs. All of the CIs reported are adjusted for design effects. All analyses were conducted using STATA (STATA Corp., College Station, TX). Economic status and residence were classified as previously described.^7^,^10^,^20^

## RESULTS

Persons who had died by follow-up were significantly older at baseline (79.5±11.9) than those who were lost to follow-up (74.3±9.1) or were successfully followed-up (74.5±8.4) (*P*<.001). A comparison of the three groups with regard to other salient features is shown in Table 1. Persons who were successfully followed up were less likely to belong to the two lowest economic classes (55.9%) than those who had died (58.0%) or were lost to follow-up (61.8). The three groups were not different with regard to sex, residence, or educational level. Those who had died by follow-up were less likely to have participated in community activities at baseline than the two other groups. The groups were not different in any other health or lifestyle features.

**Table 1:** Profile of the Sample at Follow-Up

		%			
			
Characteristic	n	Lost to Follow-Up (n = 472)	Died (n = 269)	Successfully Followed Up (n = 1,408)	*P*-Value
Sex					
Male	992	54.5	57.3	59.6	.35
Female	1,157	45.5	42.7	40.4	
Residence					
Urban	555	29.2	33.5	24.1	.17
Semiurban	870	40.0	37.5	42.6	
Rural	724	30.8	29.0	33.3	
Economic status					
Low	667	26.3	33.0	20.7	.01
Low average	763	35.5	25.0	35.2	
High average	495	26.4	26.4	29.6	
High	224	11.8	15.6	14.5	
Years of completed education					
0	1,184	53.4	48.8	54.1	.58
1–6	533	28.0	29.8	24.4	
7–12	266	10.7	12.6	13.6	
≥13	166	7.9	8.8	7.9	
Chronic medical condition					
No	1,521	73.1	66.2	70.9	.14
Yes	628	26.9	33.8	29.1	
Lifetime major depression					
No	1,598	76.7	74.0	73.7	.42
Yes	551	23.3	26.0	26.4	
Participation in household activities					
No	270	14.4	15.7	12.2	.20
Yes	1,790	85.6	84.3	87.8	
Participation in community activities					
No	279	13.7	20.1	12.2	.003
Yes	1,787	86.3	79.9	87.8	
Ever drank alcohol					
No	1,100	56.5	57.3	53.8	.44
Yes	907	43.5	42.8	46.2	
Ever smoked					
No	1,134	61.9	52.2	55.5	.22
Yes	875	38.1	47.8	44.5	

Of the 1,225 persons eligible for incidence assessment, 638 were female, and 587 were male. Of these eligible persons, 85 had developed dementia at the 3-year follow-up. With a total of 3,888 risk-years for the sample, the estimated incidence of dementia was 21.85 per 1,000 person-years (95% CI = 17.67–27.03). Women had a higher incidence (30.56/1,000 person-years, 95% CI = 23.78–39.28) than men (12.60, 95% CI = 8.44–18.80). Compared with men, the age-adjusted hazard ratio (HR) for women was 2.12 (*P* = .002). Incidence increased linearly with age such that, compared with participants aged 65 to 74, the HRs, adjusted for sex, were 2.84 (*P*<.001) for those aged 75 to 84 and 4.13 (*P*<.001) for those aged 85 and older (Table 2).

**Table 2:** Incidence of Dementia According to Sex and Age

Group	Total N	Dementia n	Sum of Risk Years	Incidence of Dementia per 1,000 Years at Risk (Exact 95% CI)	Hazard Ratio (95% CI)	*P*-Value
Total sample	1,225	85	3,888	21.85 (17.67–27.03)		
Sex						
Female	638	61	1,914.6	30.56 (23.78–39.28)	2.12 (1.32–3.41)	.002
Male	587	24	1,330.8	12.60 (8.44-18-80)	Reference	
Age						
65–74	209	16	1,914.6	9.40 (5.92–14.92)	Reference	—
75–84	358	38	1,330.8	28.55 (20.77–39.24)	2.84 (1.62–4.98)	.001
≥85	240	29	644.1	45.02 (31.29–64.78)	4.13 (2.29–7.46)	.001

CI = confidence interval.

Greater incidence of dementia was found with more-rural location of respondents' residence (Table 3). Thus, participants residing in semiurban areas had twice the risk of dementia as those residing in urban areas, and those in rural settings had a more than 150% greater risk. Participants in the low-average and low economic status groups were at significantly greater risk of incident dementia than those in the highest group. Although lack of availability of a social network, indicated by a lack of regular contacts with family and friends, was unrelated to the likelihood of new onset of dementia, poor social engagement was. Thus, persons who were not participating in family-related social activities or in community activities had twice the risk of incident dementia of those who were engaged in such activities, with the former being statistically significant.

**Table 3:** Baseline Social, Health, and Lifestyle Correlates of Incident Dementia

Correlate	Odds Ratio* (95% Confidence Interval)	*P*-Value
Residence (reference urban)		
Semiurban	2.1 (1.0–4.2)	.04
Rural	2.6 (1.3–5.2)	.01
Education, years completed (reference ≥13; some tertiary)		
7–12 (some or completed secondary)	0.7 (0.1–3.7)	.66
1–6 (some or completed primary)	0.6 (0.1–3.2)	.58
0	0.7 (0.2–3.3)	.69
Economic status (reference high)		
High average	2.9 (0.7–11.9)	.13
Low average	3.4 (1.1–11.2)	.04
Low	3.7 (1.0–14.0)	.049
Chronic medical condition (reference no)†	0.8 (0.3–2.1)	.66
Major depressive disorder (reference no)	0.7 (0.4–1.4)	.30
Mid upper arm circumference >22 cm (reference ≤22 cm)	1.3 (0.3–6.5)	.74
Physical activity (reference vigorous)		
Moderate	1.0 (0.4–2.7)	.98
Low	1.5 (0.5–4.6)	.44
Participation in household activities no (reference yes)	2.4 (1.1–5.8)	.04
Participation in community activities no (reference yes)	2.0 (0.8–5.1)	.14
Ever smoked cigarettes (reference never)		
Past smoker	0.6 (0.3–1.3)	.18
Current smoker	1.8 (0.6–5.6)	.32
Ever drank alcohol (reference no)	0.7 (0.4–1.2)	.30

* Adjusted for age, sex, and education.

† ^†^Presence of any of diabetes mellitus, hypertension, or stroke.

## DISCUSSION

This report presents incidence estimates of dementia for a cohort of elderly Yoruba Nigerians. The Yorubas are a distinct ethnic group in regard to language, culture, and factors such as diet and social organization from the other ethnic groups in Nigeria. An estimate of incident dementia of 21.85 per 1,000 person-years was obtained for the total sample. Women had twice the risk of onset, and older age was associated with greater risk of incident dementia. Of the variables assessed at baseline, rural residence, low socioeconomic status, and evidence of less social engagement were risk factors for incident dementia. None of the physical or mental health problems assessed at baseline significantly predicted dementia onset. The demographic features, including education, of the respondents correspond to the national profile for this age group.

It is important to highlight the strengths and weaknesses of the study to set its findings in context. A large cohort of elderly persons was studied and followed for an average of 39 months. The size of the sample and the duration of follow-up have yielded a reasonable number of incident cases on which to base estimates, although as in every cohort study, there was a large proportion of attrition due to deaths and loss. Persons who had died by follow-up were significantly older than those who were found and assessed, and because older age was associated with greater incidence, the reported rate may have underestimated the true incidence of dementia in the population. Tools that had been previously validated in the cultural and linguistic setting of this study were used. Even though the use of the 10-WDRT at baseline might have resulted in a substantial number of “cases” being cognitive impairment rather than dementia, the exclusion of such cases from the incidence cohort would have served to strengthen rather than weaken the estimates. At follow-up, a more-rigorous assessment procedure was used to make a diagnosis of dementia. As shown by their performance on the animal-naming fluency test, persons who received a diagnosis were clearly differentiated from those who did not, confirming the validity of the diagnosis of dementia reported here. Nevertheless, in comparing the results with those of previous studies, it is important to note that some of the differences may reflect differences in ascertainment methods.

The results presented show some important observations. First, compared to a previous study of another cohort of elderly Yoruba, the estimates reported here are higher.^4^ For example, the estimate for the group aged 65 to 74 in a previous report was 0.45% (95% CI = 0.30–0.60), whereas for the comparative group in the present report. the estimate was 9.40 per 1,000 person-years (95% CI = 5.92–14.92) (or about 0.94%). Also, in the previous study,^4^ the estimate for incident dementia for the group aged 75 to 84 was 1.69% (95% CI = 1.29–2.10), compared with an estimate of 28.04 per 1,000 person-years (95% CI = 20.32–38.70) (or about 2.8%). However, the estimate of 45.02 (95% CI = 31.29–64.78) (or about 4.5%) for the group 85 aged and older in the present report is lower than that of 5.7% (95% CI = 4.19–7.22) in the previous report. A second observation is that the overall rate of 21.85 (95% CI = 17.67–27.03) per 1,000 person-years (or about 2.2%) is considerably lower than what had been reported for African Americans (3.2%; 95% CI = 2.17–4.38), even though there is substantial overlap between the confidence intervals. Third, the overall estimates are comparable with those of many previous estimates, even though the estimates from the current study lie toward the lower end of the spectrum. Nevertheless, a closer examination of the estimates reveals other interesting observations. They are not substantially different from what have been reported for other populations younger than 80. For example, the estimate for the group aged 75 to 84 is similar to the 2.3% (95% CI = 1.5–3.6) reported previously^21^ and 22.3 (95% CI = 16.3–28.2) per 1,000 person-years for the sample in the Canadian Study of Health and Aging (CSHA).^22^ What seems to be different is the rate for persons aged 85 and older, which is lower than others have reported. Thus, for example, the rates for the groups aged 85 to 89 and 90 and older in one previous study were 8.5% and 8.2%, respectively,^21^ while the rate for those aged 85 and older in the CSHA was 106.5 per 1,000 person-years.^22^

Unlike the findings of a meta-analysis that found no sex difference in the incidence of dementia,^23^ the current study found that women had a significantly higher relative risk of incident dementia than men.

In general, factors that were found to predict incident dementia were mainly social rather than lifestyle- or health-related. Thus, more-rural residence, poorer economic status, both in a dose-response pattern, and poor social engagement significantly predicted the onset of dementia after adjusting for the effects of age, sex, and educational status. Using data from the Indianapolis-Ibadan study, an association was reported between rural living to age 19 in African Americans and incident Alzheimer's disease, and it was speculated that the mediating factor might be poverty and deprivation.^24^ Several studies have reported poor social engagements as a risk factor for incident dementia.^25^ The findings of the current study are in conformity with those of many longitudinal studies suggesting that an inverse relationship exists between the level of social engagement and the risk of dementia.^19^ The higher incidence rate in women may indicate their greater likelihood of being of low economic status and having poor social engagement. Although not significant, the association between education and incident dementia was in the opposite direction of the expectation that low education would be a risk factor. It may be that the skewed distribution of levels of education, specifically the preponderance of persons with no or few years of education in the sample, might be responsible for this trend.

It might be that these risk factors are the basis of the difference between the rates found in this larger Yoruba community than were found in the much smaller community that was the basis of the Indianapolis-Ibadan project.^7^ It is plausible that Idikan, where that earlier study was conducted, a densely populated urban community, did not offer the same level of risk of lack of social engagement that was found in a study encompassing varying levels of urban and rural environments. In general, the results suggest that, with increasing migration to urban areas of young members of the community, elderly persons residing in rural areas, especially those with low economic status, may be particularly vulnerable to less social and cognitive stimulation that may increase their likelihood of developing dementia.

## References

[1] Kalaria RN, Maestre GE, Arizaga R (2008). Alzheimer's disease and vascular dementia in developing countries: Prevalence, management, and risk factors. Lancet Neurol.

[2] Chandra V, Ganguli M, Pandav R (1998). Prevalence of Alzheimer's disease and other dementias in rural India: The Indo-US study. Neurology.

[3] Chandra V, Pandav R, Dodge HH (2001). Incidence of Alzheimer's disease in a rural community in India: The Indo-US study. Neurology.

[4] Hendrie HC, Ogunniyi A, Hall KS (2001). Incidence of dementia and Alzheimer disease in 2 communities. JAMA.

[5] Hendrie HC, Osuntokun BO, Hall KS (1995). The prevalence of Alzheimer's disease and dementia in two communities: Nigerian Africans and African Americans. Am J Psychiatry.

[6] Ineichen B (1998). The geography of dementia: An approach through epidemiology. Health Place.

[7] Gureje O, Ogunniyi A, Kola L (2006). The profile and impact of probable dementia in a sub-Saharan African community: Result from the Ibadan Study of Aging. J Psychosom Res.

[8] Osuntokun BO, Sahota A, Ogunniyi AO (1995). Lack of an association between the E4 allele of ApoE and Alzheimer's disease in elderly Nigerians. Ann Neurol.

[9] Gureje O, Ogunniyi A, Baiyewu O (2006). Apoe epsilon-4 is not associated with Alzheimer's disease in elderly Nigerians. Ann Neurol.

[10] Gureje O, Ademola A, Olley BO (2008). Depression and disability: Comparisons with common physical conditions in the Ibadan Study of Aging. J Am Geriatr Soc.

[11] Gureje O, Kola L, Afolabi E (2007). Epidemiology of major depressive disorder in elderly Nigerians in the Ibadan Study of Ageing: A community-based survey. Lancet.

[12] Welsh KA, Butters N, Mohs RC (1994). The Consortium to Establish a Registry for Alzheimer's Disease (CERAD): V-a normative study of the neuropsychological battery. Neurology.

[13] Prince M, Acosta D, Chiu H (2003). Dementia diagnosis in developing countries: A cross-cultural validation study. Lancet.

[14] Hendrie HC, Lane KA, Ogunniyi A (2006). The development of a semi-structured home interview (CHIF) to directly assess function in cognitively impaired elderly people in two cultures. Int Psychogeriatr.

[15] American Psychiatric Association, DSM-IV (1994). Diagnostic and Statistical Manual of Mental Disorders.

[16] Kessler RC, Ustun TB (2004). The World Mental Health (WMH) Survey Initiative version of the World Health Organization (WHO) Composite International Diagnostic Interview (CIDI). Int J Methods Psychiatr Res.

[17] Chilima DM, Ismail SJ (1998). Anthropometric characteristics of older people in rural Malawi. Eur J Clin Nutr.

[18] World Health Organization (1999). WHO-Disability Assessment Schedule II.

[19] Craig CL, Marshall AL, Sjöström M (2003). The International Physical Activity Questionnaire (IPAQ): A comprehensive reliability and validity study in twelve countries. Med Sci Sports Exerc.

[20] Ferguson BD, Tandon A, Gakidou E, Murray CJL, Evans DB (2003). Estimating permanent income using indicator variables. Health Systems Performance Assessment: Debates, Methods and Empiricism.

[21] Paykel ES, Huppert FA, Brayne C (1998). Incidence of dementia and cognitive decline in over 75s in Cambridge: Overview of cohort study. Soc Psychiatry Psychiatr Epidemiol.

[22] The Canadian Study of Health and Aging Working Group (2000). The incidence of dementia in Canada. Neurology.

[23] Gao S, Hendrie HC, Hall KS (1998). The relationship between age, sex, and the incidence of dementia and Alzheimer disease: A meta-analysis. Arch Gen Psychiatry.

[24] Ogunniyi A, Hall KS, Gureje O (2006). Risk factors for incident Alzheimer's disease in African Americans and Yoruba. Metab Brain Dis.

[25] Fratiglioni L, Paillard-Borg S, Winblad B (2004). An active and socially integrated lifestyle in late life might protect against dementia. Lancet Neurol.

